# Remarks on the Pathology of Tuberculosis

**Published:** 1870-12

**Authors:** John C. Peters


### Remarks on the Pathology of Tuberculosis.

BY JOHN C. PETERS, M. D.

In accordance with the desire of the President that all the Fellows, or as many as possible, of the Academy, should prepare themselves for a discussion of the so-called new views of tubereulosis, I have made some preparation.

This was the more easy, as I had paid somewhat particular attention to the subject in 1854, and again in 1850—and more or less, perhaps less, since then.

I find nothing very new, if we admit all the facts and theories of Virchow and Niemeyer, and even add still more to them, derived from the labors of such excellent physiological chemists as Simon of Berlin, Lehman of Leipzig, and Day of St. Andrews College, Edinburgh. They are simply a return to the notions of Carswell, that tuberculosis commences in the air cells and smaller bronchi; and to those of Addison, Broussais, Wilks, etc., that it generally arises from some form of inflammation of a low type; and to those of Hasse, Preuss, Cerrutti, Vogel, Guterbock, etc., that tubercle is always caseous.

Even if we side with Virchow and Niemeyer, it is not yet necessary to modify seriously the doctrines of Laennec and Louis, or those of Swett, Clark or Flint, or the opinions of the mass of the profession in this and every other country.

This arises principally from the fact, that our views of the nature and treatment of inflammation are very diffeaent from what they were in the times of Laennec and Broussais; and it may easily be made very clear that it would have been a great misfortune, from the times of Laennec almost down to the present day, if Laennec had adopted Broussais's, or even Niemeyer's actions of inflammation, provided he had carried them out to the full extent that Broussais had impressed upon them.

In our times, which are those of Niemeyer, it makes very little difference whether we lean to the theory of the subacute inflammatory, or dyscratic origin of tubercle. We treat all fevers and inflammations very differently now from what Broussais did of old, and the treatment of tuberculosis is not very seriously modified by Niemeyer, simply because he has adopted the modern or restorative treatment of inflammation.

It also makes very little difference whether we cling to the idea that there is only one form of tubercle, or admit that there are two. For true miliary tubercle often undergoes the caseous degeneration, and primary caseous deposits are often followed by the secondary formation of the miliary tubercles, as is proven by the experience of every practical physician who is in the habit of seeing or making post-mortem examinations; and we believe that both the miliary and tuberous forms of tubercle are made up of casein.

Every observing physician must have seen cases of true general miliary and tuberculosis occurring in almost every organ, viz.: in the membranes of the brain, in the lungs, pleura, spleen, peritoneum, kidneys, etc., and yet not preceded by any caseous or yellow tubercle that could be discovered.

Again, every physician must have seen cases of primary and chronic scrofulous or tuberculous disease of the glands of the neck, followed by miliary tubercles of the brain, lungs, etc., not attended by any caseous or yellow tuberous tubercle, or mass, n any external organ that could be discovered. The two diseases precede or follow each other indiscriminately, because they are closely allied to each other, if not absolutely identical.

These remarks may seem all the more strange because I strongly incline to some of the so-called modern views of tuberculosis, except I think them very old.

To make these points more clear, Dr. Peters exhibited to the members of the Academy thirty-three colored plates of portions of lung, showing tubercolosis acuta vel miliaris; pneumonia catarrhalis of children; Niemeyer's catarrhal pneumonia; pneumonia ulcerosa; connective tissue corpuscles; cancer formed in connective tissue like pus, and tubercle; tubercle granules in air-cells from Bennett, 1858; tubercle from lung tissue, normal pus globules, tuberculous matter with melanosis, etc.,—all from Dr. Swett, 1852; bronchitis with both plastic and cheesy deposit, expectoration in hepatization, congestion, etc.,—from Dr. Dobell; blood globules in phthisis from Thompson; deposits of Tubercle both inside and outside of the air-cells and bronchi, etc.

1st. As to the question whether there are one or two forms of tubercle.

The majority of the profession are now inclining to the opinion that there is but one elementary form of tuberculous deposit, viz.: the gray granulation, or miliary tubercle; and that many of the cases in which extensive and uniform cheesy deposits are found in the lungs depend more upon chronic catarrhal, or a low and unhealthy type of the inflammatory process, or even scrofulous inflammation of the part, than upon true tuberculous formation.

We have to decide whether these latter are simply imflammatory or truly tuberculous—or whether they may become one or the other. My own opinion is that there is no difference between the two forms of the disease, except their location and size, and that large yellow tubercles are always mixed with the debris of epithelium, mucus, pus, exudation-globules, etc., in the lungs.

Virchow throws no light upon the point why miliary tubercle is formed in the connective tissue; but this substance is one of the few membranes of the body which contain *casein.* On the authority of Lehman of Leipzig, and Day of Edinburgh, it may be stated that casein exists normally in the expressed juice of the connective and elastic tissues. And here we hit, perhaps, upon the first link in the long chain of facts which go to prove the connection of caseous degeneration with tubercle; and we almost immediately find another, viz.: it frequently happens that the albumen in the parenchymatous fluid or plasma which moistens and nourishes all the tissues, assumes a *casein-like* character; *i. e.*, it does not coagulate on heating, is precipitated by dilute acetic acid, and separates in the form of a superficial membrane on evaporation. Hence the fluid which bathes the connective tissue, and nourishes the connective tissue corpuscles, always is somewhat, and may easily become abnormally *caseous.*

These points may also throw so e light upon the fact that true miliary tubercle is commonly developed in the adventitia or lamina of connective tissue immediately surrounding the blood vessels. We know that the parenchymatous fluid which moistens and nourishes the tissues transudes through the capillaries—and if that be already too albuminous, or even caseous, a cacoplastic or cheesy material will be poured out into the connective tissue, and may excite it to increased growth and proliferation; each minute newly formed granule or cell being made up of caseous material, instead of the more gelatinous and fibrous substance which goes to form true healthy cellular or connective tissue.

But cheesy transformation is not peculiar to tubercle; for it is well-known that extravasated blood may undergo it; effusions of all kinds, both exudative and inflammatory; epithelium newly formed cells, connective tissue corpuscles; and all the albuminoid constituents of the blood, such as albumen, fibrin, globulin, etc.; pus, too, often becomes cheesy, and even cancer and sarcoma occasionally. Hence the time seems to have arrived when we must perforce admit a caseous or cheesy transformation or degeneration of many tissues and exudations, just as we have long ago admitted a fatty degeneration, an amyloid transformation, a fibroid conversion, and saccharine, lardaceous, and waxy metamorphoses.

This theory of caseous degeneration has been progressing slowly in the minds of the profession for many years. Over twenty-five years ago Rokitansky was teaching that many abnormal products and exudations, which were not absolutely and evidently tubercular at first, soon began to tuberculize and assume the appearance of yellow or caseous tubercules.

For a long number of years before 1857, the celebrated Dr. Addison, of Guy's hospital, was strenuously opposing the idea that all or much of the deposit found in phthisical lungs should be called tubercle.

Addison's successor, Dr. Wilks, Lecturer on Pathology and Curator of the Museum of Guy's Hospital, continued from and after 1857 to teach that soft, yellow, caseous matter was often undoubtedly produced without being preceded by crude tubercle, and was merely a degenerative change, which even simple substances of a fibrous or cellular character might pass into. He thinks, however, that miliary tubercles are quite distinct in formation and character, and not merely in location and size, from the soft yellow tubercle; but correctly states it is this latter product which is most important in phthisis (simply because it occurs in larger masses and greater quantities.) He even goes so far as to assert that, in speaking of the ordinary disorganizing processes seen in phthisis, he will entirely dismiss miliary tubercle from consideration, and look to the (caseous) material which he thinks is all essential in the various phases of the disease.

We all know that our Dr. Sweet, who is certainly held in affectionate remembrance by many who are here, taught long before 1833 that tubercles were generated in pleuritic exudations and adhesions, and he often tells us in his writings that we will frequently meet with tuberculous disease of the lungs which has followed an attack of the pleurisy, and is secondary to it.

The views of these men, and of Virchow, Reinhart, Vilemin, and others, made but little impression upon the profession at large, until Niemeyer sprang almost like a harlequin into the medical arena, declaring that the greatest danger for the majority of consumptives is, that they are apt to become tuberculous.

We now propose to examine whether we may or must admit a caseous dyscrasia or degeneration of many tissues, products, exudations, cells, &c., just as we have long ago accepted the reality of fatty degerations, amyloid degenerations, saccharine transformations, and lardaceous and waxy changes.

The best chemical analysis of tubercle quite distinctly corroborate some of the so-called modern views; and they have this great advantage, that they were made long before any theory was at stake upon their results. Thus the accurate Hasse, who drew his conclusions from his own experiments, and from numerous analyses carefully compiled from Cerutti and Vogel, found the organic component parts of tubercle to consist principally of *casein*, with some little fat and albumen.

As regards casein, we know that the primitive food of all young mammals, viz., milk, contains casein, which is readily transformed into albumen of the brain, liver, kidneys, and other albuminous organs; and into the musculo-fibrin, or syntonin, which go to make up the extensive muscular system; and we now know that these substances are often transformed back again into casein.

It is true Virchow thinks that the casein of milk is formed from the epithelial cells which line the lacteal ducts. These are supposed to be made in enormous quantities during lactation, are transformed partly into casein, and partly into fat, and then are generally swept out by the gushing tide of fluids pushing behind them. Occasionally they are plastered and occluded in the milk-ducts, just as caseous or quasi-tuberculous deposits often are in the air-cell and smaller bronchi, which, it is well known, are abundantly supplied with epithelium as the milk ducts.

Casein is also a highly sensitive substance to chemical reagents, often undergoing decided changes from the application of the mildest of them.

We know that gelatine, albumen, and fibrin may be formed out of casein, and we are also sure that the major part of the tissues and parenchyma of the various organs are made up out of gelatine, albumen and fibrin; but none of them are composed of unchanged casein. Casein is a foreign and inimical substance to them. Hence we are almost justified, at this early stage, in concluding that disease may arise from the formation and deposit of a cacoplastic lymph, largely made up of casein, or at least of one which soon, and readily undergoes caseous degeneration, and that this constitutes the essence of scrofula and tubercle.

If we look for the origin of this caseous substance, our attention is at first drawn to the globulin of the blood-corpuscles. These globules contain nearly 300 parts in 1,000 of globulin and cell membrane; and globulin is a substance approaching so nearly in properties to casein, that the great Berzelius called globulin the casein of the blood. The almost equally well-known physiological chemist, Schmidt, also calls hæmato-globulin blood-casein; and our Dr. Draper says, globulin is a substance approaching so nearly in properties to casein, that we may without much presumption, infer that it can easily be converted into it.

Now we know that in the circulating blood there are continuous endosmotic currents between the serum, or so-called intercellular fluid, and the viscid red liquid which occupies the interior of the blood cells. Hence we are almost prepared to concur with Panum, Moleschott, Has, etc., that casein is a normal constituent of the serum of the blood, and to infer that it may occur in an excessive quantity in the tuberculous or some other dyscrasia.

Again, it is well known that the iron of the blood is deficient in phthisis; and it is equally true, though not equally well-known, that a considerable quantity of the fatty matter contained in the blood is contained in the blood-globules, which in their dry state should contain, according to Lehman, from two to three per cent of fat. Hence, if the globulin of the blood-globules contains too much casein and too little fat, the normal transudations and the abnormal exudations may easily undergo a cheesy degeneration.

As the predominating ingredients of the blood-globules are globulin, fat, and hæmatin, and all of these are defective and deficient in tuberculous blood, we would naturally expect to find some alteration or defect in the shape and conformation of the blood-globules in phthisis. The well-known Dr. Theophilus Thompson, Clinical Lecturer at the Brompton Hospital for Consumptives, has paid particular attention to this point, and I show his drawings of the alterations which the blood-globules undergo in phthisis.

It is well to add, that in phthisis the red globules may diminish in number from 130 to 78, and the white globules be much increased, so that a peculiar state of the blood, resembling that which obtains in leuchæmia, or immediately succeeds digestion, when any lymph-globules have been quickly poured into the blood, is often found in phthisis.

If the blood-globules are defective in phthisis, so is the serum of the blood. We have already seen that too much casein and too little fat is returned to it from the blood-globules.

The water and albumen of the serum are also in excess, and the albumen is defective in quality, being less capable of being oxidized or transformed into fibrin, and doubtless more easily convertible into casein. According to Aitkin, this defective albumen in the blood of phthisical persons may increase from 76 to 100 parts in one thousand.

In phthisis we also have a blood which is less alkaline than it should be: the serum may be so feebly alkaline that it often becomes turbid from the presence of very small dark molecules of a protein body, because the albuminate of soda in the blood has been deprived of some of its alkali, and a portion of the albumen thus freed from its soda separates in a molecular form.

Finally, it is equally well known that the normal fluid of the blood-globules naturally contains a great preponderance of the potash salts and phosphates, while plasma or serum has the greater part of the soda salts, especially the phosphate and chloride of sodium—all these are deficient, and this accounts for the feeble alkalinity of the blood in phthisis.

To sum up:—The albumen and water of the serum are in excess in phthisis.

The fat and iron are deficient.

The alkaline salts are deficient.

The serum-casein is in excess.

When this state of the blood obtains, a too caseous serum may be supplied to the connective tissue, followed by the caseous degeneration and proliferation that Virchow places so much stress upon; and thus miliary tubercles may be formed.

Or a caseiform, too albuminous, or too little plastic or fibrinous substance may exude silently and slowly, in a very insidious manner, into the air-cel s and smaller bronchi, etc., and yellow caseous tubercle may arise.

Or, under the influence of a slight or severe cold, or bronchitis, or catarrhal pneumonia, exudations may take place in the lungs, air-cells and bronchi, which may quickly undergo the yellow tuberculous or caseous degeneration, together with the epithelium ,mucous and exudation corpuscles, and other substances found in them.

Thus we may have the combined local and constitutional origin oi tubercle.

Away back, as far as the times of Baillie, tubercular disease was supposed to arise from an infiltration of an excessively albuminous fluid, of a thick or synovial-like character, which then gradually degenerated into a fine granular mass, which also included portions of lung tissue when it softened down into pus.

And we have seen the so-called modern views really differ very little from this; if we substitute casein for albumen, all the rest of the theory may remain nearly unchanged.

The correct treatment of tuberculosis flows easily and naturally from the above views. Fatty substances; alkaline remedies, especially the phosphates of soda and potash; iron, especially in the form of the phosphate of iodide, and the avoidance of an excess of albuminous and caseous substances, especially skimmed milk, must always form the bases of our remedial means.—*Med. Record.*

